# Preoperative therapy with sunitinib malate in a patient with a gastrointestinal stromal tumor and liver metastases: A case report

**DOI:** 10.1097/MD.0000000000014222

**Published:** 2019-12-20

**Authors:** Zhi-Qiang Wang, Zheng-Qi Wen, Jun Yang, Hong-bin Zhang, Zhi-yong Kou, Rui-Ze Zhou, Wen-Liang Li

**Affiliations:** a Department of General Surgery, The Second Hospital of Tianjin Medical University, Tianjin; b Department of Oncology, The First Affiliated Hospital of Kunming Medical University, Kunming, China.

**Keywords:** gastrointestinal stromal tumors, liver metastases, preoperative therapy, sunitinib malate

## Abstract

**Rationale:**

Patients with gastrointestinal stromal tumors (GISTs) are often found to have liver metastases at their 1st presentation. Most patients need preoperative treatment to reduce the size of the liver metastases to increase the possibility of surgical resection. Currently, imatinib mesylate is the drug of 1st choice for preoperative treatment and sunitinib malate (SM) is seldom used. Here we report a case of GIST with liver metastases where SM was used as a preoperative treatment.

**Patient concerns:**

A 56-year-old worker presented with intermittent abdominal pain and eating difficulties.

**Diagnoses:**

An enhanced computed tomography scan showed a 15 × 15 × 10 cm malignant mass in the upper abdomen, and 2 metastases (15.1 × 13.1 cm and 14.8 × 8.8 cm) in the liver. The postcaval and middle hepatic veins were compressed by the liver metastases, making radical resection very difficult.

**Interventions:**

First the primary tumor in the jejunum was resected, and then SM was used as a preoperative treatment to reduce the size of the liver metastases to improve the possibility of surgical resection.

**Outcomes:**

Both liver metastases regressed considerably in size and it was then possible to perform a radical resection.

**Lessons:**

The SM has the potential to be used as preoperative therapy for GIST with large liver metastases. This method provides a new option for the preoperative treatment of GIST with liver metastases.

## 1 Introduction

At present, surgery is the preferred treatment of gastrointestinal stromal tumor (GIST). Surgery combined with postoperative targeted therapy significantly improves the overall survival and progression-free survival time of patients with GIST.^[1–3]^ DeMatteo et al^[4]^ reported that liver metastases could be resected in only 26.0% (34/131) of patients with GIST with liver metastases, and the other patients needed to undergo preoperative therapy with imatinib mesylate (IM) to achieve an opportunity for surgical treatment. Related reports show that 50% of the patients with GIST with liver metastases or recurrence received complete excision after IM treatment.^[5]^ Furthermore, after preoperative therapy with IM, in about 13% of the patients with GIST with unresectable liver metastases complete excision could then be achieved. Some doctors^[6]^ consider the use of tyrosine kinase inhibitors combined with multiple surgical excision was the optimal treatment to improve overall survival.

The target sites of sunitinib malate (SM) include VEGFR1-3, CD117, KIT, PDGFRA, and PDGFRb. The active spectrum of SM is wide, and it can inhibit mutations causing IM resistance and significantly prolong the survival time of advanced patients with GIST who had failed to respond to IM therapy.^[7]^ Here, we report a case in which surgery was combined with treatment with SM to treat a jejunal GIST with irresectable liver metastases.

## 2 Case presentation

**Figure 1:**
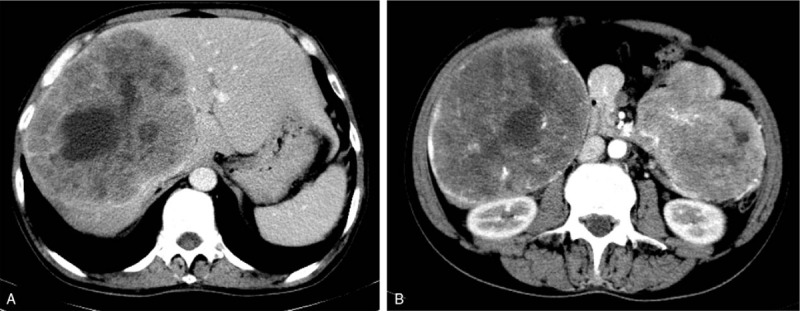
Tumor size (longest diameter × shortest diameter) in computed tomography (preoperative). (A) 12.4 × 14.5 cm. (B) Liver 12.8 × 10.7 cm. Intestinal tumor 10.5 × 7 cm.

A 56-year-old worker presented with 5 months of intermittent abdominal pain and eating difficulties. We performed an abdominal enhanced computed tomography (CT) scan and the results revealed the presence of a 15 × 15 × 10 cm malignant mass in the upper abdomen, and 2 metastases (15.1 × 13.1 cm and 14.8 × 8.8 cm) in the liver (Fig. 1). The postcaval and middle hepatic veins were compressed by the liver metastases, making radical resection of the metastases very difficult.

This study was conducted at the First Affiliated Hospital of Kunming Medical University and the procedures used were approved by the Ethics Committee of the First Affiliated Hospital of Kunming Medical University. The procedures conformed to the tenets of the Declaration of Helsinki, and informed written consent was obtained from the patient for the treatment and for publication of this case report and accompanying images after a full explanation of the procedures to be used and the possible complications.

During the operation, we found the primary tumor located in the 1st segment of the jejunum, about 10 cm distal to the Treitz ligament. The primary tumor of the jejunum was removed at the operation. The postoperative pathological report was as follows: GIST of jejunum, mitotic count 3/50 HPF, CD117(+), CD34(+), Dog-1(+), Ki67 index 30%, exons 11 mutation. The risk of recurrence of the GIST was high. It is well known that IM is the best choice as 1st-line therapy for patients with the exon 11 mutation, but eventually SM was chosen for this patient because of serious adverse drug reactions of IM. The treatment protocol used for SM was 50 mg taken orally once daily, for 4 consecutive weeks followed by a 2-week rest period (schedule 4/2) comprising a complete cycle of 6 weeks.

**Figure 2:**
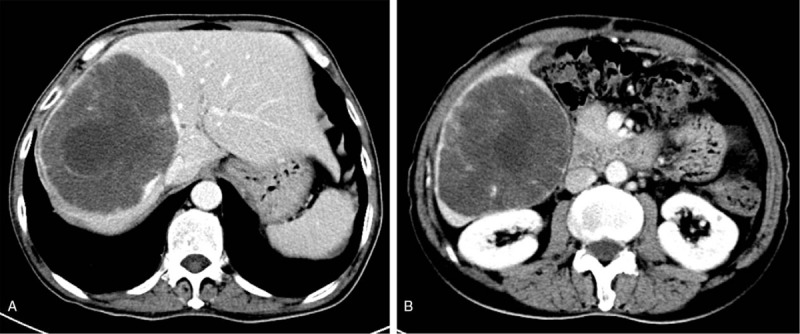
Tumor size (longest diameter × shortest diameter) on computed tomography (1 month after operation). (A) 10.3 × 13.2 cm. (B) 11.3 × 9.6 cm.

**Figure 3:**
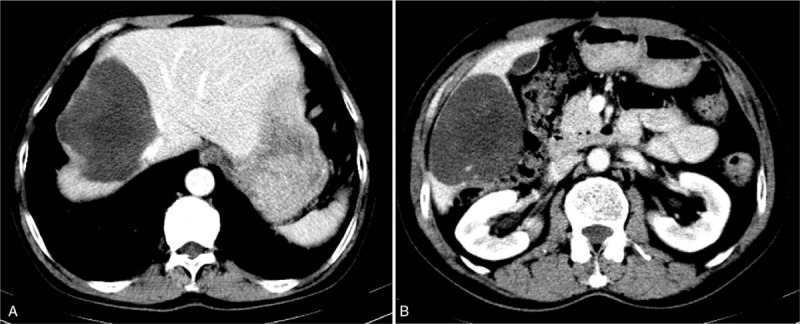
Tumor size on computed tomography (1 year after operation): the multiple liver lesions were smaller than before. (A) 8.4 × 10.3 cm. (B) 9.4 × 7.3 cm.

One month after the surgery, the abdominal enhanced CT scan, was repeated and we were surprised to find that the liver metastases in the right lobe had obviously regressed, indicating that SM was effective (Fig. 2). At this time, the patient had only taken SM for 2 weeks. One year later, the liver metastases had become much smaller. There was a clear distance between the liver metastasis and the middle hepatic vein. Moreover, postcaval vein compression had been alleviated (Fig. 3).

**Figure 4:**
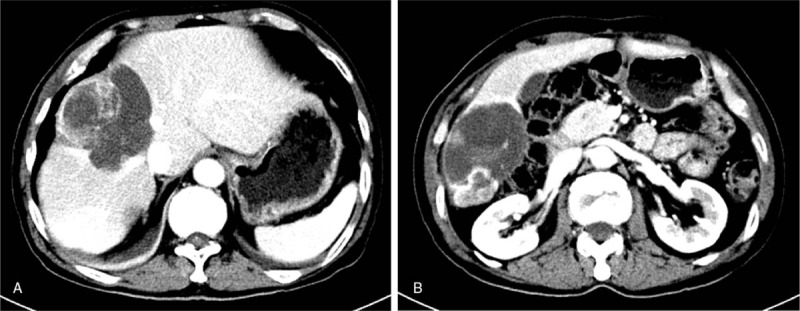
Tumor size on computed tomography (32 months after operation): multiple solid portions of the liver mass were increased. (A) 8.4 × 8.5 cm. (B) 9.1 × 6.3 cm.

Two years and 8 months later, an enhanced CT scan indicated that the size of the liver metastases had now increased (Fig. 4), indicating that resistance to SM had now occurred; therefore, the patient underwent a right hemihepatectomy.

**Figure 5:**
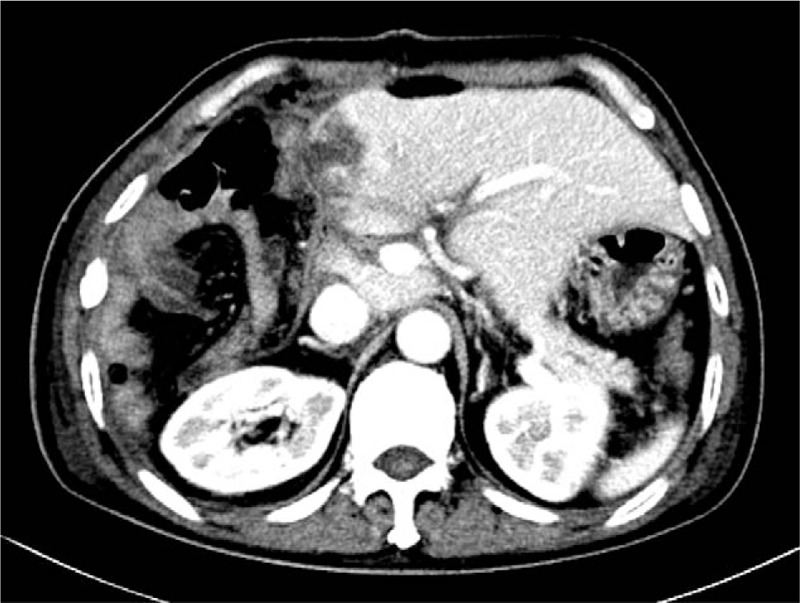
Computed tomography: the liver after right hemihepatectomy. Postoperative recovery was uneventful.

The postoperative pathologic report was as follows: GIST liver metastasis, CD117(+), CD34(+), and Dog-1(−), exons 11 and 17 mutation. IM was administered instead of SM. One month later, CT results indicated that neither recurrence nor metastases were detectable (Fig. 5).

## 3 Discussion

Because the residual liver volume was not enough and the synchronous liver metastases were irresectable, we only resected the primary tumor of the jejunum during the 1st operation. The pathologic result of the primary tumor was GIST and with an exon 11 of c-kit mutation. At present, SM is used as a 2nd-line treatment for patients with GIST with IM intolerance or resistance. However, using SM as preoperative therapy for patients with GIST with liver metastases to make surgical removal of the metastases has seldom been reported. Because of serious adverse drug reactions of IM the patient was given SM to try and achieve the possibility of resection of the liver metastases. To our surprise, the liver metastases regressed quickly and significantly; neither local recurrence nor any new metastases occurred while the patient was taking SM. Preoperative therapy with SM obtained a satisfactory result, consistent with the report of Kikutake et al.^[8]^

According to the current National Comprehensive Cancer Network (NCCN) guidelines of soft-tissue sarcoma, preoperative treatment of IM can be continued for 6 to 12 months until the maximal response has been achieved. In this case, 1 year after the patient started to take SM, the liver metastases in the right lobe showed obvious regression There was then a clear distance between the liver metastasis and the middle hepatic vein. Moreover, postcaval vein compression was alleviated. The patient had gained the best opportunity for resection of the liver metastases. From this case, we can infer that preoperative treatment with SM can continue for as long as 1 year. However, the patient refused surgery due to fear of the operation. Eventually, 2 years and 8 months later the liver metastases had progressed and the patient was persuaded to accept surgery and the liver metastases were successfully removed. Because of the delay we believe that the patient had missed the best window of opportunity for surgical treatment. If the patient had accepted surgery and removal of the liver metastases when it was 1st suggested, the progress of the disease could have been greatly delayed. Because the liver metastases had started to increase in size after 2 years and 8 months of control by SM we believed that SM resistance had occurred, and the patient was advised to begin treatment with IM after he underwent right hemihepatectomy.

In this case, the preoperative therapy of the liver metastases with SM caused obvious regression and we obtained an opportunity for surgical resection. This showed that SM has the potential as a preoperative therapy for unresectable liver metastases. This method provides a new possibility for the preoperative treatment of GIST complicated by liver metastases. Whether SM can be used as a preoperative therapy of unresectable liver metastases needs to be verified by further studies.

## Author contributions

**Conceptualization:** Zhi-Qiang Wang, Wen-Liang Li.

**Data curation:** Zhi-Qiang Wang, Zheng-Qi Wen, Jun Yang.

**Formal analysis:** Zhi-Qiang Wang, Hong-bin Zhang, Zhi-yong Kou, Rui-Ze Zhou.

**Writing – original draft:** Zhi-Qiang Wang.

**Writing – review & editing:** Zhi-Qiang Wang, Wen-Liang Li.
